# Exploring the Barriers to and Motivators for Using Digital Mental Health Interventions Among Construction Personnel in Nigeria: Qualitative Study

**DOI:** 10.2196/18969

**Published:** 2021-11-09

**Authors:** Janet Mayowa Nwaogu, Albert P C Chan, John A Naslund, Carol K H Hon, Christopher Belonwu, Jackie Yang

**Affiliations:** 1 Department of Building and Real Estate The Hong Kong Polytechnic University Kowloon Hong Kong; 2 Department of Global Health and Social Medicine Harvard Medical School Boston, MA United States; 3 School of Civil Engineering and Built Environment Queensland University of Technology Queensland Australia; 4 Gribs Integrated Services Limited Port Harcourt Nigeria

**Keywords:** mental health, construction personnel, digital technology, digital intervention, barriers, motivators, mobile phone

## Abstract

**Background:**

Work-related stress in the construction industry increases the prevalence of depression and anxiety among personnel. In low-resource settings such as Nigeria, construction personnel face high demands and severe working conditions but only have a few services to address their mental health needs. With emerging research showing that digital interventions can be used to self-manage mental health across diverse settings, there may be new opportunities to support construction personnel in the construction industry.

**Objective:**

This study aims to determine the use of digital interventions for mental health management among construction personnel in Nigeria and to explore the factors that facilitate or impede the use of these interventions.

**Methods:**

This qualitative study explored the perspectives of a convenience sample of 62 construction personnel. The data were subjected to inductive content analysis.

**Results:**

A total of 6 barrier and 3 motivator themes were identified and categorized into 2 groups. The barrier themes were subcategorized into barriers to adoption and barriers to persistent use, whereas the motivator themes were subcategorized into intrinsic and extrinsic motivators. Lack of awareness and knowledge about the interventions may constitute a barrier to adoption and use. Participants frequently reported concerns regarding their effectiveness and usability.

**Conclusions:**

This study provides an understanding of the design needs required to facilitate sustained self-management of mental health based on the experiences and expectations of construction personnel with digital interventions.

## Introduction

### Background

Construction personnel are reported to have a high incidence of common mental health problems [1-3], which results from high levels of stress owing to the nature of their work and working conditions. These include poor remuneration [4], working in extreme temperatures [5], long working hours, job insecurity, role ambiguity [6], demanding jobs [7], work-life imbalance [8], and organizational injustice [9,10]. In the Netherlands, the prevalence rates of depression and posttraumatic stress disorder symptoms among construction supervisors were 18% and 20%, respectively, whereas among skilled workers the rates were 11% and 7%, respectively [11]. In Nigeria, the prevalence rates of depression, anxiety, and suicidal ideation among construction supervisors were 55.1%, 14.8%, and 9.2%, respectively, whereas among tradesmen the rates were 74.5%, 36.4%, and 14.6%, respectively [12,13]. Generally, the weighted prevalence rates of depression, anxiety, and suicidal ideation in Nigeria were 5.5%, 3.5%, and 7.28%, respectively [14,15]. As untreated mental health problems pose a significant risk for suicidality [16] and other adverse health effects, there is an urgent need for mental health management among personnel in the construction industry and allied professions.

For lower middle-income countries such as Nigeria, there has been an increase in attention toward personnel’s mental health and well-being needs, with recent efforts to mitigate stress reactions such as depression among construction personnel [4,10,17,18]. As the burden of mental health problems is predicted to increase over time [19], with an estimated 100 million mobile broadband users and 149 million active Global System for Mobile Communication subscribers in Nigeria, the country ranks highest in the internet market in Africa [20]; therefore, leveraging digital technology may yield more flexible, cost-efficient, and convenient methods to support the mental health of construction personnel. For instance, technology has proven to be potentially useful in reducing health management costs and challenges associated with mental health problems [21]. Through digital technologies such as smartphones, there may be opportunities for workers to manage stress responses and seek support for their mental health concerns when needed [1,22], while reducing possible barriers and stigma associated with accessing face-to-face services delivered on-site.

Workplace mental health interventions (eg, secondary and tertiary interventions) are used to mitigate and treat mental ill health [23] and overcome barriers to the use of psychological therapies delivered in mental health facilities. However, such workplace interventions are limited due to the need for in-person treatment, barriers to receiving and accessing care, stigma, shortage of mental health professionals, and high cost of service [1,24,25]. This is especially relevant among employees in the construction sector, as their roles are often mobile and involve excessive work hours [1]. Digital mental health interventions designed to meet specific needs, for example, managing stress, mood, anxiety, mental fitness, and well-being delivered through digital technologies such as smartphones, wearables, websites, or computers provide scalable interventions to improve health and health care [26].

Despite the surge in apps and websites, digital mental health interventions are underutilized [27]. A reason for the low adoption of interventions in mental health management in the workplace may be due to commercially available interventions that are not best suited to the workplace setting, user needs, and context [1]. In addition, these interventions often suffer from an abrupt exit by users without achieving the intended health goals [28]. To promote a sustained use of digital interventions until the desired outcome is achieved, identifying barriers and motivators for continued use is crucial. Such insights can inform changes that may be needed for the design and efficacy of the intervention; without that, designers will largely rely on intuition [29]. This means identifying barriers to and motivators for the use of digital mental health interventions will impart valuable design information to support the development of efficient and user-friendly interventions [30].

### Barriers and Motivators

Stiles-Shield et al [29] used card-sorting tasks to evaluate the barriers to using and engaging smartphone apps in managing depression among 20 potential users in the United States. The identified barriers to use were concerns about efficacy and functionality (ie, issues about effectiveness, misfit of feature to need, and crashing of the app), data privacy, insufficient feedback, and cost of data plans. Carolan et al [27] studied barriers to and facilitators of user engagement in a digital intervention aimed at psychoeducation and stress management among employees recruited from different sectors. Using semistructured interviews, the study deduced that the facilitators of digital intervention use and engagement included content, design, motivational emails (ie, quotes and advice), information on well-being, confidentiality, and support from the organization.

The use of digital interventions in construction industries, particularly wearable technologies for health and safety, has been studied [31,32]. However, these studies neither geared toward secondary intervention nor explored barriers to and motivators for use on an individual basis. Studies on occupational health have considered the barriers to and motivators for using digital mental health interventions among male-dominated professions in Australia [22,27]. Nonetheless, there remains a dearth of literature regarding high-risk populations, such as construction personnel in Nigeria. It is also unknown what constitutes the barriers and motivators to use such interventions among them. There is limited information on how construction personnel in low-resource countries use commercially available interventions to manage their mental health, what forms of barriers and motivators they experience, and whether their desired recovery goals are achieved.

### Objectives

Examining users’ experiences in specific contexts with digital mental health interventions could provide new information to better design future interventions. It is unknown how construction personnel in a low-income country such as Nigeria use existing digital mental health interventions to manage their health and what forms of barriers and motivators they encounter. Therefore, this study aims to fill the existing gap by exploring the satisfaction of construction personnel with publicly available digital interventions for mental health management in Nigeria. To achieve this aim, the following objectives were set: (1) to determine the barriers to use and engagement with digital mental health interventions among construction personnel in Nigeria and (2) to determine the motivators to use these interventions.

The ability to ensure engagement with the interventions after uptake will potentially allow construction personnel to cope with work stress and enhance positive mental health, especially if appropriate engagement theories and user needs ground the development of such interventions. This can inform future design improvements, enabling acceptability and sustained use to achieve the intended mental fitness and health goals. This study adds to a limited body of knowledge by describing the factors that influence the probable uptake and use of digital mental health interventions by construction personnel in a lower middle-income setting. This study defines barriers as reasons or obstacles that prevent the use of digital interventions for mental health management. At the same time, motivators refer to catalysts for use, purpose, or factors that enhance the application or desire to use a digital mental health management intervention [33].

## Methods

### Theoretical Background

The Unified Theory of Acceptance and Use of Technology (UTAUT2) was chosen as the framework to guide this study as it is robust in incorporating previous acceptance models and is considered a highly sensitive model for explaining consumers’ variance in the use of technology [34,35]. The UTAUT2 was adopted in this study through a bottom-up inductive approach to aid the interpretation of results and guide the design of future digital interventions. The UTAUT2 incorporates 7 constructs (ie, performance expectancy, effort expectancy, social influence, facilitating conditions, hedonic motivation, price value, and habit) and 4 moderators (ie, age, gender, experience, and voluntariness) [36]. The first 5 constructs of the UTAUT2 relate to the enjoyment derived from using a product [37].

### Sample and Measurement

This study used an open-ended survey, a qualitative technique. This technique was applied as it is often used to solicit feedback on products and design needs [38,39]. In the digital intervention literature, qualitative techniques are widely used as part of formative research efforts aimed at gaining insights from users about intervention design, content, and engagement [38]. Similarly, the qualitative technique was used to elicit feedback on digital interventions for mental health management from construction industry end users.

This technique is suitable as it provides a broader picture of the subject and provides an avenue for participants to express their concerns and feedback about the topic [40]. Such feedback can inform future intervention designs for the industry, whereby the industry can leverage such technologies to maintain mental fitness and promote good mental health among its workforce. Construction personnel engaged in supervisory and monitoring roles in Nigeria formed the target population for this survey and were determined based on a convenience and snowball sampling method.

The respondents included site supervisors (ie, site engineers) and project managers based on a log collected from the Nigerian Institute of Building. These groups of construction personnel were considered for 3 reasons: (1) involvement in building production management; (2) ease of eliciting the required information; and (3), should the needs of the construction personnel be met, leveraging digital interventions for universal prevention and secondary mental health interventions across the entire construction workforce would be easier. The research methodology involved 2 steps: (1) survey questions were drafted based on literature reviews and (2) a pilot study was conducted with industry practitioners. The Mobile Application Rating Scale, a validated and reliable scale used for assessing the quality of health apps [41], was also used in this study. As this study explored the use of digital mental health interventions and not only apps, these questions were adapted for use in this study.

The survey questions were written in English Language and elicited demographic characteristics such as age, gender, and years of experience. Other information obtained was related to the type of digital mental health intervention that participants may have used, length of use, the intended goal of the intervention, and barriers to and motivators for the use of digital interventions. The questionnaire also contained a statement regarding informed consent and data confidentiality. As this survey pertained to adoption, satisfaction, and dissatisfaction with digital interventions used for mental health management, detailed information about the profession of respondents was not required. Before administering the questionnaire, the survey questions were piloted and reviewed by a professor with >15 years’ experience in various aspects of occupational health and safety and a public health professional with expertise in health promotion and mental health. The Human Subjects Ethics Sub-Committee of Hong Kong Polytechnic University approved this study.

Piloting was performed to conduct face and content validity, assess readability, clarify language, and establish the appropriateness of the developed content within the intended context of use [42]. Suggestions made included soliciting information on gender, years of experience, and social media use, whereas some questions were reworded. The final survey is presented in Multimedia Appendix 1 [27,41,43,44]. The survey questions were then developed using Google Forms, and the link was sent via an email to the respondents using Google Docs and the Mail Merge add-on by Quickluition.

### Data Collection

The questionnaire was disseminated to the construction personnel via their organizational or personal email addresses. The Mail Merge add-on for Google Docs facilitated sending bulk and personalized emails to the respondents. An email introducing the study and containing the link to the survey was sent to the professionals. The first page of the survey link contained informed consent and confidentiality statements. On the basis of convenience sampling, a total of 335 emails were sent to the construction personnel in Nigeria between December 2018 and July 2019, while they were also encouraged to send out the survey link to their colleagues engaged in on-site building production and management.

A total of 62 responses were received following the convenience and snowball sampling. By responding to the questionnaire, this implies that 62 respondents gave informed consent. However, the number of responses were comparable with studies by Carolan and de Visser [27] and Anderson et al [43] on digital health interventions based on 18 and 22 responses, respectively. Comparable response rates (10%-20%) are common for surveys conducted using emails or web-based mediums [45]. Although it is difficult to ascertain the total number of surveys sent out and the corresponding response rate due to the snowball sampling, Bowen et al [46] recorded a response rate of 7% following a web-based survey on work stress, stress effect, and coping in the construction industry.

The low number of responses may also be attributed to the research area being mental health management and due to stigma, it is possible that potential respondents thought that digital mental health interventions are meant only for persons with mental ill health symptoms. Responses were obtained from the respondents using Google Forms. To protect the confidentiality of respondents, the survey questions did not elicit contact details and affiliation of respondents in the survey form, and all responses collected through Google Forms were kept anonymous.

### Data Analysis

Inductive content and thematic analysis of the retrieved data were supported using MAXQDA version 18.1.1 software for mixed methods analysis developed by VERBI GmbH. To ensure the reliability of the data analysis, the themes and categories were drawn in 2 stages to avoid bias; the codes, themes, and categories were also checked and agreed upon by at least 3 of the authors. First, the context or interpretation of the data was later refined using themes in the existing literature. The word cloud, word frequency, phrase finder, and categorization of survey data features were used as appropriate for supporting content and thematic analysis.

The word cloud and frequency tools were used to identify the most used words; the font size of such words was bold compared with others [47]. After identifying the most used words, they were read in line with the sentences they appeared in and autocoded appropriately. This implies, for instance, when asked, “Do you use social media? If yes, kindly mention those you mostly use.” The responses included “yes, Facebook, WhatsApp, Linkedin” (Respondent NG #1); “FB, read news and latest post from my friends” (Respondent NG #3). On performing the analysis, words such as “Facebook, WhatsApp, LinkedIn, FB, and, read, news” were identified. The words were then read in line with the sentences in which they appeared to determine their worth. For example, “and, read, news” were not autocoded, whereas other words were autocoded. “FB” was also coded to “Facebook.”

To avoid bias, the phrase finder (word combination) was used to identify possible themes and autocoded for longer sentences. Finally, to check for mistakes and refine the coding, the *categorize survey data* was used, after which refinement was performed manually. All autocoded themes in each question were refined in line with themes from previous studies using the *categorize survey data* tool. Subsequently, all segments were edited appropriately following the themes identified from previous literature or using a new theme as relevant to the statements. Concluding on themes during autocoding or refinement was also done with reference to the sentence in which they appeared as well as themes identified in Stiles-shield et al [29] and Simblett et al [30], followed by the descriptive analysis. The codes before refinement are shown in Multimedia Appendices 2 and 3.

## Results

### Overview

Of the 62 respondents, 56 (90%) were male and 6 (10%) were female, and the mean age was 40 (SD 13.18) years. All respondents owned a smartphone and were active social media users. When asked which social media platforms they mostly used, respondents indicated that they used Facebook, WhatsApp, Instagram, Twitter, LinkedIn, and Telegram. Of the 62 respondents, only 24 (39%) had ever used digital interventions for mental health management. This implies that less than half of the respondents used their mobile devices or computers for issues related to their mental health.

Information was sought on the type of digital mental health interventions used by the respondents (Table 1). It was deduced that the interventions used included mobile-based apps (14/24, 58%) and web-based apps (10/24, 42%). It was further revealed that the respondents used mental health interventions for stress, depression, anxiety management, and mental fitness. In addition to using digital mental health interventions, 26% (16/62) of the respondents reported using activity or fitness trackers and other wearable technology. The wearable technologies used include activity trackers and bracelet technology aimed at maintaining mental health and fitness through health and wellness interventions.

**Table 1:** Demographics and how construction personnel in Nigeria use digital interventions for their mental health (N=62).

Demographic information			Participants, n (%)
**Work experience (years)**			
	1-5		18 (21)
	6-10		18 (24)
	11-20		12 (21)
	>20		14 (33)
**Respondents who use electronic gadgets (mobile devices or computers) for mental health–related issues**			
	Yes		24 (39)
	No		38 (61)
**Respondents who use digital interventions for their mental health (n^a^=24)**			
	**Type of mental health intervention used**		
		Mobile-based app	14 (58)
		Web-based app	10 (42)
**Respondents who use wearables (n=16)**			
	**Type of wearable devices**		
		Activity trackers	15 (91)
		Bracelet technology	1 (9)
**Wearable technology connection or synchronization (n=16)**			
	Smartphone connected		15 (91)
	Standalone		1 (9)
**Aim or purpose of using such digital intervention (n=17)**			
	Anxiety		6 (25)
	Depression		5 (21)
	Stress management		4 (17)
	Mental fitness		2 (8)

^a^n: the number of responses.

The wearables were mostly phone connected or synchronized, although a few others, including the bracelet, were standalone wearables. The activity or fitness trackers include different brands of Fitbit, Apple Watch, Smartwatch, and Garmin mediums, used for lifestyle interventions, including physical activity, sleep tracking, heart rate, and blood pressure monitoring.

### Barriers to Using Digital Interventions for Mental Health Management

When asked, “If you do not use your gadgets for anything concerning your mental health, would you like to?” Among the 38 participants who were yet to use such interventions, 28 (74%) expressed willingness to use their electronic gadgets for mental health management. This signaled that barriers to the adoption of digital mental health interventions among the participating construction personnel in Nigeria could be related to a lack of awareness or limited knowledge about digital mental health interventions. In addition, of the 28 respondents willing to adopt digital mental health interventions, 4 (14%) indicated needing them for depression and anxiety management.

Respondents mentioned dislikes about the interventions and reasons to stop use, highlighting barriers to the continual use of digital mental health interventions (Table 2). Of the 24 respondents who had used their gadgets for something concerning their mental health, 15 (63%) reported no dislikes that will cause them to stop using the interventions, whereas 9 (37%) expressed some dislikes and reasons for stopping use. These dislikes and reasons for discontinuing digital mental health interventions were grouped into 5 categories: usability, efficiency and effectiveness, high cost, boring due to lack of human interface, and time-consuming.

**Table 2:** Linking the findings to the Unified Theory of Acceptance and Use of Technology (UTAUT2) framework.

Construct, themes					Responses		Identified UTAUT2 construct
**Barriers**							
	**Barrier to adoption**						
		Lack of awareness and knowledge		—^a^		N/A^b^	
	**Barriers to persistent use**						
		Usability		Navigation problems due to having too many options Quite complicated Technical malfunction Battery life Synchronization demands		Effort expectancy, facilitating conditions	
		Efficiency and effectiveness		Not logical or practical Not correct Not accurate or not efficient To obtain a better one		Performance expectancy	
		High cost		Subscription cost Data usage		Price value	
		Boring due to lack of human interface		Robotic in nature and boring		Facilitating conditions	
		Lack of time		Time consuming to input answers to all questions asked		Effort expectancy	
**Motivators**							
	**Intrinsic and extrinsic**						
		Efficiency and effectiveness		It is OK (ie, good) It gives correct answers Good and better mental health (ie, improved health) Serve the purposes for which it is meant for Give accurate results when monitoring health status Stress reduction relaxes palpitations reduces stress Quick access to health monitoring quick access to solution provides fast access to monitor mental ill health symptoms		Performance expectation Performance expectancy Performance expectancy, social influence	
		Usability		Easy to use App working well		Facilitating conditions, effort expectancy	
	**Extrinsic**						
		Providing motivations		Client’s prompt motivational pull sessions		Hedonic motivation	

^a^Not available.

^b^N/A: not applicable.

Feedback from respondents on the barriers to the persistent use of digital mental health interventions includes the following:

I use an app for mood tracking, quite good, but the options make it quite complicated. The wearable device I use can be annoying due to of the synchronization time. I need to delete initial sync to the app sometimes and start all over, and it can be stressful.

Respondent #2

The applications have too many options, navigation becomes too tedious.

Respondent #22

It is sometimes not correct. The beginning question on checking depression seems like prepared to classify me into having depression always. Sometimes, I input the wrong answers, but it still says I have depression. Sometimes, I struggle with downtimes, but not always.

Respondent # 41

The app works but some of the activities to relieve stress don’t seem to be logical or practical to help the situation.

Respondent #13

I use a paid app for my stress and anxiety, while it seems good for me and needs dedication, subscribing is a bit expensive because it is international, maybe a local content will be a bit cheaper.

Respondent #34

It is so time-consuming. It can consume time as someone has to answer several questions.

Respondent #53

Respondents noted they would stop using the interventions for the following reasons:

It was consuming my data, so I stopped using it.

Respondent #8

It is expensive to maintain premium subscription on the app I use.

Respondents #34

I want to get a more accurate one. I used a free one that is artificial intelligence empowered; it asked questions about stress and how my sleep was. I answered by typing how unrested I feel when I wake, and it said I understand, followed by, that is, a lot of questions. The reply was off point. It put me off. I stopped using immediately.

Respondent #41

It is robotic, nobody to respond or communicate. It can be boring. It is not exact.

Respondent NG #14

### Motivators for Using Digital Interventions for Mental Health Management

To determine the motivators for using digital mental health interventions, information was elicited on likes for interventions and services or things that would boost continual use of the interventions (Table 1). Furthermore, additional features that users of mobile-based apps would like to see in future mental health apps were determined. Motivators are measures that would support customer satisfaction and engagement. These features could also facilitate the continued use of mobile-based applications and reduce premature dropout from digital interventions when used in mental health management.

When asked what they liked about the interventions, participants’ responses were grouped into 2 categories (Table 2): *efficiency and effectiveness* and *usability*. Efficiency and effectiveness were also related to the ability of the interventions to aid stress reduction and quick access to health monitoring. Specifically, respondents indicated the following:

It is quite easy to use, though the questions are not straightforward.

Respondent #1

Feedback from instructor.

Respondent #12

Gives correct answers.

Respondent #17

I used it to monitor my anxiety level.

Respondent #16

It makes me quickly check up on what level I am at my mental health space, fast access to checking the level of depression symptoms, though sometimes not completely accurate.

Respondent #41

Quick access to solution.

Respondent #22

It reduces stress.

Respondent #21

It relaxes me from palpitations

Respondent #27

Respondents were asked what services or features would encourage them to continually use the interventions until the desired health goal was achieved. The respondents indicated that ensuring the *usability*, *efficiency and effectiveness* of the interventions, and their ability to *provide motivational pulls* would boost sustained use. Respondents highlighted the following services or features to encourage engagement:

An app that works well.

Respondents #13

Accurate results, it should be more accurate.

Respondents #18

If it serves the purpose to which it is meant, then it is worth continuing.

Respondents #22

Client’s prompt motivational pull sessions.

Respondents #12

When asked about additional features to incorporate into a digital mental health app, respondents indicated music, educative posts, motivational posts (ie, quotes), news alerts, comedy skits, and other health features, for example, some respondents mentioned the following:

Educative posts on health and some on work.

Respondent #1

Meditational music.

Respondent #45

Music.

Respondent #53

I want an app that will be more than just mental health, an Artificial Intelligence-based app that is educative on other stress-related issues.

Respondent #58

## Discussion

### Principal Findings

This study shows that key barriers to using digital mental health interventions among construction personnel in Nigeria include efficiency and effectiveness, usability, high cost, boring due to lack of human interface, and lack of time. On the basis of these results, lack of awareness of and knowledge about digital mental health interventions, if not attended to, may constitute a barrier to adoption. Hence, the barriers can be categorized as barriers to adoption and barriers to persistent use. Regarding the motivators to use, respondents mentioned usability, efficiency and effectiveness, and motivational features as important reasons they liked the interventions or features that could boost user engagement. It is evident that these are foundational characteristics that digital interventions need to possess to ensure user engagement and persistent use. Owing to the shortage of studies on this topic among lower middle-income economies and construction personnel, comparisons made in the discussion are drawn from evidence in the general body of knowledge, especially from populations in high-income countries.

### Barriers to and Motivators for Using Digital Mental Health Interventions

#### Performance Expectancy

Performance expectancy measures were related to both barriers and motivators to using the interventions among construction personnel. They included efficiency and effectiveness measures, such as giving a better result, practicability to meet desired mental fitness and health needs, stress reduction ability, and quick access to health monitoring. In the case where the expectations were not met, it appeared a barrier to use, whereas if otherwise, it was a motivator for continued use. The ability of these interventions to enhance stress reduction and quick access to health monitoring can be described as an intrinsic motivator for using them. Generally, people use their digital devices to conduct a web search for information about their mental health, understand their experiences, and learn strategies to cope effectively [48]. These digital interventions allow for regular health assessments and quick access to resources for health-related information [49]. They can also be efficient and easy to use [50].

Meeting efficiency and effectiveness need is critical, as health care consumers are more likely to adopt and use electronic health technologies that can deliver the intended goals. In this study, some respondents had to stop using mental health apps due to efficiency and effectiveness concerns. There is a general concern over the ability of digital mental health interventions to meet intended treatment needs [29]. It is essential to conduct thorough quality testing for all features or services before deployment to facilitate engagement with digital mental health interventions. A crucial step to meeting effectiveness demand is for developers of future digital mental health interventions to adequately address the outlined concerns and needs that constitute barriers to use.

Bakker et al [51] suggested that such efficiency and effectiveness concerns could be linked to a lack of experimental validation for many publicly available digital mental health interventions. The ineffectiveness of the interventions may have resulted from a one-size-fits-all approach, which makes digital interventions less flexible and personalized [52]. Future digital interventions for the construction industry and allied professions in lower-middle-income settings such as Nigeria would benefit from ensuring that the gold standard for validation, which is a randomized controlled trial, is followed to ensure efficacy.

#### Facilitating Conditions and Effort Expectancy

Similarly, facilitating conditions and effort expectancy needs were both barriers and motivators to use. The facilitating conditions construct was related to usability and feelings that the interventions are boring due to lack of human interaction and personalized feedback, whereas concerns about usability and lack of time were related to effort expectancy. Usability appeared as a motivator and barrier. This finding is consistent with previous research, including a study that showed that boredom while using digital interventions to manage health negatively impacts user engagement [43]. To solve concerns about the lack of feedback, the integration of human support in interventions is a potentially valuable strategy [29] and may assist in eliminating the robotic feeling and perception of ineffectiveness. Digital interventions for mental health delivered on the web (ie, web-based) that incorporate human guidance or coaching appear to yield better results and user engagement than unguided interventions [27,29]. A significant obstacle to the use of electronic health services by potential users is the absence of support or resources, which would allow them to use such services properly [35].

Usability concerns were related to a technical malfunction, which may have resulted from a sudden failure in the functionality of mobile apps. In this case, mental health apps were reported to fail during use, thus restricting the proper use of the interventions. Difficulties arising from connecting wearable devices with apps, the disappearance of apps (from the digital interface or dashboard), system freezing, and loss of power are significant factors that can affect the usability of digital interventions in managing health [30]. Another factor that affected the usability of digital mental health interventions was navigation demands, as some interventions were reported to have too many navigation options, making them complex to use.

Ensuring usability cannot be overlooked because construction personnel are more likely to use interventions that are easier to grasp when managing mental health [35]. To address usability concerns in mental health apps, it is important that apps are user tested and determined to be of good quality before deployment. A detailed guide for proper installation and use can also be provided to solve the functionality, synchronization, and navigation demands. It was also deduced that a large amount of time required to input information on feelings into digital mental health interventions was worrisome. This finding was consistent with that of Peng et al [44] in that the time required to input responses on feelings can be reduced by putting measures in place to satisfy the effort expectancy construct.

#### Price Value

This study reveals that the price value construct of UTAUT2 was related to high costs. High cost was identified as a barrier, as respondents mentioned that the cost of subscribing to mental health–related apps and internet access were deterrents for use. It included dislike for the cost involved in subscribing for the paid version of mental health apps as most were from a context outside Nigeria and involved elevated exchange costs and the cost for internet data. Furthermore, the cost of internet access (ie, data package) for deploying mental health apps [29] is a consistently reported barrier to using such interventions to manage mental health. Consumers of a product usually consider the cost related to acquiring a product or service; thus, price value is an essential determinant of use and acceptance [35]. Therefore, context-based interventions guided by strong psychological principles should be developed for a lower middle-income country such as Nigeria and its construction industry to address high cost concerns. Designers should ensure that efficiency and effectiveness are maintained while meeting affordability. This would mean that although extra (secondary) features can differ between subscription packages, the primary features necessary for effective mental health management should be the same and meet the required standard.

Most apps require a web-based connection, especially in cases of linked services, such as listening to meditation music. It would be necessary that designers include a download button, to enable downloading real-time services such as meditation music or e-coach session over a wireless network, which can be followed when a user is no longer connected to wireless access. This is especially important in low-resource settings such as Nigeria, where wireless connectivity can be highly variable. Designers should address these barriers in future digital interventions designed for the Nigerian context, as this would help ensure sustained engagement with digital interventions toward reaching the desired health goal and the promotion of improved mental health.

#### Hedonic Motivation

The need for quick motivational pull-ups and feedback could also serve as important extrinsic motivators and help solve the negative impact of social influence. For example, taking web-based interventions delivered through social media groups. Although such forums have helped in mental health management, concerns about the quality of information and materials accessed through such platforms have been highlighted [48]. The role of motivating users of such interventions is critical and should not be taken for granted. Integrating quick motivational pulls and feedback can increase their effectiveness and use. For online support groups, moderators should quickly attend to the concerns or issues raised. This will also help ensure efficiency and effectiveness, offering more significant potential to boost adoption and engagement.

#### Desired Additional Features for Mental Health Apps

Respondents indicated that comedy skits, music, educational posts, motivational posts, and news were additional features that they would like to see incorporated into future mobile-based apps. There is evidence showing that to increase employees’ engagement with digital mental health interventions, motivational quotes are often sought [27]. Carolan et al [27] reported that half of the participants in their study affirmed receiving motivational quotes and well-being information, preferably every Monday morning. Such motivational messages are indicated to improve mental health, as people who choose to receive them show significant symptom reduction [53].

In the case where such interventions are personalized for delivery in an industrial setting, educational (informative) posts relating to occupation, health, and well-being can potentially improve engagement. The desire for comedy skits was a recurrent feature desired among the respondents and beams light in the context of Nigeria. The respondents reiterated that this feature, along with its effectiveness, would facilitate continual use. Wilkins et al [54] noted that humor and laughter impart health and well-being. In the Nigerian context, comedy skits are a popular medium of humor, which generates laughter that helps mitigate depression and other stress reactions [55]. Agwu and Tiemo [54] reported that laughter and biofeedback are ways to cope with stress among construction personnel. Comedy is a big business in Nigeria, a menu served at nightclubs in Nigeria and has moved from stand-up to web-based skits [55,56]. This may be because they provide a way to distract Nigerians from the worsening socioeconomic conditions that stare them in the face. Incorporating animated comedy skits related to stress, mental health, and well-being as an additional feature in future mental health apps can provide hedonic motivation, thereby facilitating persistent use while delivering its primary health goals.

Designers of digital interventions will need to use their discretion to determine whether it is practicable to incorporate these additional features into an intervention while targeting the primary outcomes of mental health management. Mobile app interventions for mental health promotion of construction personnel can be enhanced with motivational posts (ie, quotes), educational posts, comedy skits, and preloaded relaxation music. Additional user feedback should be obtained, and the effectiveness of these features should be validated before deployment.

### Limitations

Despite the overall sample size being small, it was valuable for an exploratory study of this nature. It is important to note that these results act as a guide to inform future studies focused on developing and delivering digital mental health interventions in the construction industry in a lower-middle–income country. Efforts were made to solicit as many responses as possible. However, because it was a web-based survey without compensation for respondents, many individuals may not have been self-motivated to respond. Another reason might be that the study is related to mental health, and individuals may have declined to participate due to stigma issues or misconceptions about mental health or cultural beliefs prevalent in Nigeria. The reduced response rate could also be due to the perception of no previous or intended need for such digital interventions for mental health management.

Another limitation of this study was the use of an anonymous web-based survey and open-ended questionnaire, which did not allow a follow-up to probe into some responses. However, this methodology was adopted to ensure anonymity and explore diverse views among users within the Nigerian construction industry. Owing to the limited sample size, it was not possible to determine the influence of age and gender on digital intervention use and acceptability, as detailed by UTAUT2. Nevertheless, because this study was exploratory and not conclusive, the influence of the variables was not a necessary factor. Finally, owing to the size of this study, the results may not be generalizable to other low-resource countries, similar occupational groups, or construction men. Therefore, these results should be interpreted with caution. However, this study spurs research reasoning in this direction.

### Conclusions

This study explored the use of digital mental health interventions for self-management among construction personnel in Nigeria. Several barriers and motivators to the use of digital interventions in mental health management were deduced from this study and linked to the UTAUT2 framework following the inductive content analysis process. The barriers comprised usability, efficiency and effectiveness, high cost, boring due to lack of human interface, and lack of time. The motivators were efficiency and effectiveness, usability, and motivation.

This study shows that efforts are needed to provide awareness of the use of digital interventions for mental health management among construction personnel. This awareness should emphasize the different outcomes related to such interventions, including stress management, adequate sleep management, and mental fitness, which would help maintain good health. The construction industry needs to leverage digital interventions to ensure the mental fitness of its workers. For instance, cognitive behavioral therapy is also a preventive technique and can be delivered through digital mental health interventions, thus helping to prevent mental ill health. Therefore, the adoption of digital mental health interventions will be a potentially important avenue for delivering mental health services and universal prevention interventions throughout the construction industry in low-resource settings.

Although most of the findings are consistent with previous findings on digital health interventions, this study has extended the body of knowledge on digital interventions for mental health management by exploring construction personnel in Nigeria. The study reported that low awareness and knowledge about digital mental health interventions may be a viable barrier to use, with stress reduction (ie, the ability to reduce stress) as an intrinsic motivator to use. Furthermore, salient features desired by study respondents as additions to mental health apps included comedy skits, music, educative posts (resources), and motivational posts (ie, quotes). The respondents did not want their apps to be just a health tool; they also desired it as an educational and motivational tool. Future research should examine the suitability of such educational resources, such as routine tips on health, safety, and well-being. The provision of motivational quotes would be more impactful if they are enhanced with personalized features and tailored to the needs of unique users. Tailoring has proved to be highly effective in supporting health communication and education efforts [44].

Future research should examine how organizations can adequately influence the use of digital mental health interventions in the Nigerian construction industry without fear of stigmatization. This study has revealed that feedback from users of digital mental health interventions can inform design information. This exploratory study offers a first step toward identifying some of the barriers to and motivators for the use of digital interventions in lower middle-income countries such as Nigeria. The next step would be to conduct a quantitative survey based on the findings of this study, which will then allow for comparative analysis and more conclusive results to inform the field of research. There is a need to further examine the level of awareness and knowledge about digital mental health interventions among construction personnel in Nigeria. This will help determine how uptake is affected by the knowledge about the different mental health fitness abilities that such interventions hold.

The findings from this study suggest that attending to these barriers and motivators will assist in eliminating abrupt exits from digital interventions for mental health management. The ability to ensure engagement with the interventions will allow construction personnel to cope with work stress and enhance positive mental health, especially if appropriate engagement theories and user needs ground the development of such interventions. The results of this study will assist in the design of new digital mental health interventions to deliver to personnel in the construction industry and improve existing interventions. Therefore, there is a need to leverage digital mental health interventions within the Nigerian construction industry for mental health promotion.

## Appendix

Multimedia Appendix 1 Complete survey question.

Multimedia Appendix 2 Initial themes on motivators based on the likelihood of using digital interventions in mental health management.

Multimedia Appendix 3 Initial themes based on responses to barriers to using digital interventions in mental health management.

## Abbreviations

UTAUT2: Unified Theory of Acceptance and Use of Technology

## References

[1] Deady M, Johnston DA, Glozier N, Milne D, Choi I, Mackinnon A, Mykletun A, Calvo RA, Gayed A, Bryant R, Christensen H, Harvey SB (2018). A smartphone application for treating depressive symptoms: study protocol for a randomised controlled trial. BMC Psychiatry.

[2] Sunindijo R, Kamardeen I (2017). Work stress is a threat to gender diversity in the construction industry. J Constr Eng Manage.

[3] Roche AM, Pidd K, Fischer JA, Lee N, Scarfe A, Kostadinov V (2016). Men, work, and mental health: a systematic review of depression in male-dominated industries and occupations. Saf Health Work.

[4] Oladinrin TO, Adeniyi O, Udi MO (2014). Analysis of stress management among professionals in the Nigerian construction industry. Int J Multidiscipl Curr Res.

[5] Ibem EO, Anosike MN, Azuh DE, Mosaku TO (2011). Work stress among professionals in building construction industry in Nigeria. J Construct Econom Build.

[6] Yip B, Rowlinson S (2009). Job burnout among construction engineers working within consulting and contracting organizations. J Manage Eng.

[7] Leung M, Liang Q, Olomolaiye P (2016). Impact of job stressors and stress on the safety behavior and accidents of construction workers. J Manage Eng.

[8] Lingard H, Francis V, Turner M (2012). Work time demands, work time control and supervisor support in the Australian construction industry. Eng Const Arch Manag.

[9] Chih Y, Kiazad K, Cheng D, Capezio A, Restubog Sl (2017). Does organizational justice matter? Implications for construction workers’ organizational commitment. J Manage Eng.

[10] Bowen P, Edwards P, Lingard H (2013). Workplace stress experienced by construction professionals in South Africa. J Constr Eng Manage.

[11] Boschman JS, van der Molen HF, Sluiter JK, Frings-Dresen MH (2013). Psychosocial work environment and mental health among construction workers. Appl Ergon.

[12] Nwaogu JM, Chan AP, Tetteh MO (2021). Staff resilience and coping behavior as protective factors for mental health among construction tradesmen. J Eng Design Technol.

[13] Nwaogu JM (2021). An integrated approach to improve mental health among construction personnel in Nigeria. Thesis and Dissertations - The Hong Kong Polytechnic University, Hong Kong.

[14] Adewuya AO, Atilola O, Ola BA, Coker OA, Zachariah MP, Olugbile O, Fasawe A, Idris O (2018). Current prevalence, comorbidity and associated factors for symptoms of depression and generalised anxiety in the Lagos State Mental Health Survey (LSMHS), Nigeria. Compr Psychiatry.

[15] Adewuya AO, Ola BA, Coker OA, Atilola O, Zachariah MP, Olugbile O, Fasawe A, Idris O (2016). Prevalence and associated factors for suicidal ideation in the Lagos State Mental Health Survey, Nigeria. BJPsych Open.

[16] Li H, Luo X, Ke X, Dai Q, Zheng W, Zhang C, Cassidy RM, Soares JC, Zhang XY, Ning Y (2017). Major depressive disorder and suicide risk among adult outpatients at several general hospitals in a Chinese Han population. PLoS One.

[17] Ojo GK, Adeyeye GM, Opawole A, Kajimo-Shakantu K (2019). Gender differences in workplace stress response strategies of quantity surveyors in Southwestern Nigeria. Int J Build Pathol Adapt.

[18] Sorensen G, Nagler EM, Pawar P, Gupta PC, Pednekar MS, Wagner GR (2017). Lost in translation: the challenge of adapting integrated approaches for worker health and safety for low- and middle-income countries. PLoS One.

[19] Charles CN, Msagati T, Swai H, Chacha M (2019). Microalgae: an alternative natural source of bioavailable omega-3 DHA for promotion of mental health in East Africa. Scientific African.

[20] Russon MA (2020). How internet access is improving in Nigeria. BBC News.

[21] Mohr DC, Duffecy J, Jin L, Ludman EJ, Lewis A, Begale M, McCarthy M (2010). Multimodal e-mental health treatment for depression: a feasibility trial. J Med Internet Res.

[22] Deady M, Peters D, Lang H, Calvo R, Glozier N, Christensen H, Harvey SB (2017). Designing smartphone mental health applications for emergency service workers. Occup Med (Lond).

[23] Joyce S, Modini M, Christensen H, Mykletun A, Bryant R, Mitchell PB, Harvey SB (2016). Workplace interventions for common mental disorders: a systematic meta-review. Psychol Med.

[24] Dickerson FB, McNary SW, Brown CH, Kreyenbuhl J, Goldberg RW, Dixon LB (2003). Somatic healthcare utilization among adults with serious mental illness who are receiving community psychiatric services. Med Care.

[25] Inkster B, Sarda S, Subramanian V (2018). An empathy-driven, conversational artificial intelligence agent (Wysa) for digital mental well-being: real-world data evaluation mixed-methods study. JMIR Mhealth Uhealth.

[26] Murray E, Hekler EB, Andersson G, Collins LM, Doherty A, Hollis C, Rivera DE, West R, Wyatt JC (2016). Evaluating digital health interventions: key questions and approaches. Am J Prev Med.

[27] Carolan S, de Visser RO (2018). Employees' perspectives on the facilitators and barriers to engaging with digital mental health interventions in the workplace: qualitative study. JMIR Ment Health.

[28] Nahum-Shani I, Hekler EB, Spruijt-Metz D (2015). Building health behavior models to guide the development of just-in-time adaptive interventions: a pragmatic framework. Health Psychol.

[29] Stiles-Shields C, Montague E, Lattie EG, Kwasny MJ, Mohr DC (2017). What might get in the way: barriers to the use of apps for depression. Digit Health.

[30] Simblett S, Greer B, Matcham F, Curtis H, Polhemus A, Ferrão J, Gamble P, Wykes T (2018). Barriers to and facilitators of engagement with remote measurement technology for managing health: systematic review and content analysis of findings. J Med Internet Res.

[31] Aryal A, Ghahramani A, Becerik-Gerber B (2017). Monitoring fatigue in construction workers using physiological measurements. Auto Construct.

[32] Hwang S, Lee S (2017). Wristband-type wearable health devices to measure construction workers' physical demands. Auto Construct.

[33] Passey ME, Longman JM, Robinson J, Wiggers J, Jones LL (2016). Smoke-free homes: what are the barriers, motivators and enablers? A qualitative systematic review and thematic synthesis. BMJ Open.

[34] Heselmans A, Aertgeerts B, Donceel P, Geens S, Van de Velde S, Ramaekers D (2012). Family physicians' perceptions and use of electronic clinical decision support during the first year of implementation. J Med Syst.

[35] Tavares J, Oliveira T (2017). Electronic health record portal adoption: a cross country analysis. BMC Med Inform Decis Mak.

[36] Venkatesh V, Thong J, Xu X (2016). Unified theory of acceptance and use of technology: a synthesis and the road ahead. J Asso Inform Syst.

[37] Idoga PE, Toycan M, Nadiri H, Çelebi E (2019). Assessing factors militating against the acceptance and successful implementation of a cloud based health center from the healthcare professionals' perspective: a survey of hospitals in Benue state, northcentral Nigeria. BMC Med Inform Decis Mak.

[38] Singer E, Couper MP (2017). Some methodological uses of responses to open questions and other verbatim comments in quantitative surveys. MDA J Quant Methods Survey Methodol.

[39] Vitale DC, Armenakis AA, Feild HS (2008). Integrating qualitative and quantitative methods for organizational diagnosis. J Mixed Methods Res.

[40] Miller AL, Dumford AD (2014). Open-ended survey questions: item nonresponse nightmare or qualitative data dream?. Surv Pract.

[41] Stoyanov SR, Hides L, Kavanagh DJ, Zelenko O, Tjondronegoro D, Mani M (2015). Mobile app rating scale: a new tool for assessing the quality of health mobile apps. JMIR Mhealth Uhealth.

[42] Afolabi MO, Bojang K, D'Alessandro U, Ota MO, Imoukhuede EB, Ravinetto R, Larson HJ, McGrath N, Chandramohan DH (2014). Digitised audio questionnaire for assessment of informed consent comprehension in a low-literacy African research population: development and psychometric evaluation. BMJ Open.

[43] Anderson K, Burford O, Emmerton L (2016). Mobile health apps to facilitate self-care: a qualitative study of user experiences. PLoS One.

[44] Peng W, Kanthawala S, Yuan S, Hussain SA (2016). A qualitative study of user perceptions of mobile health apps. BMC Public Health.

[45] Fricker Jr RD (2008). Sampling methods for web and e-mail surveys. The SAGE Handbook of Online Research Methods.

[46] Bowen P, Govender R, Edwards P (2014). Structural equation modeling of occupational stress in the construction industry. J Constr Eng Manage.

[47] Fielding J, Fielding N, Hughes G (2012). Opening up open-ended survey data using qualitative software. Qual Quant.

[48] Bucci S, Schwannauer M, Berry N (2019). The digital revolution and its impact on mental health care. Psychol Psychother.

[49] Wetterlin FM, Mar MY, Neilson EK, Werker GR, Krausz M (2014). eMental health experiences and expectations: a survey of youths' web-based resource preferences in Canada. J Med Internet Res.

[50] Naslund JA, Aschbrenner KA, Scherer EA, McHugo GJ, Marsch LA, Bartels SJ (2016). Wearable devices and mobile technologies for supporting behavioral weight loss among people with serious mental illness. Psychiatry Res.

[51] Bakker D, Kazantzis N, Rickwood D, Rickard N (2016). Mental health smartphone apps: review and evidence-based recommendations for future developments. JMIR Ment Health.

[52] Scholten H, Granic I (2019). Use of the principles of design thinking to address limitations of digital mental health interventions for youth: viewpoint. J Med Internet Res.

[53] Whitton AE, Proudfoot J, Clarke J, Birch MR, Parker G, Manicavasagar V, Hadzi-Pavlovic D (2015). Breaking open the black box: isolating the most potent features of a web and mobile phone-based intervention for depression, anxiety, and stress. JMIR Ment Health.

[54] Wilkins J, Eisenbraun AJ (2009). Humor theories and the physiological benefits of laughter. Holist Nurs Pract.

[55] Adesoye RE (2018). Phonological distortion as a humorous strategy in Folarin Falana’s comedy skits. Eur J Humour Res.

[56] Adetunji A (2013). The interactional context of humor in Nigerian stand-up comedy. Pragmatics.

