# Supplementary material for: Exploring the Barriers to and Motivators for Using Digital Mental Health Interventions Among Construction Personnel in Nigeria: Qualitative Study
Source: JMIR Form Res. 2021 Nov 9;5(11):e18969. doi: 10.2196/18969 (PMC8663629; doi:10.2196/18969)
Supplement: Multimedia Appendix 1 [file formative_v5i11e18969_app1.docx]

Multimedia Appendix 1**:** Complete survey question

| Question | Option | Source | Response per question |
| --- | --- | --- | --- |
| Gender |  |  | 62 |
| Years of work experience in the construction industry |  |  | 62 |
| Age | 25-34; 35-44; 45-54; 55-64 |  | 62 |
| Is your cellphone a smartphone? | Yes, No |  | 62 |
| Do you use social media? | Yes, No |  | 62 |
| If yes, kindly mention those you mostly use |  |  |  |
| NB: Interventions refer to digital technology help, products, or services used to self-manage their mental health. | | |  |
| Do you ever use your mobile phone or other devices for anything related to your mental health? |  | [1] | 24 |
| If No, please would you be interested in using digital technologies (mobile phones or devices) for your mental health? |  |  | 28 |
| If you use a mobile mental health intervention, what kind of intervention is it (for example, app-based, online-based, etc.)? |  |  | 24 |
| Do you use any wearable devices (such as wrist wearables, activity trackers, etc.)? If yes, please mention which wearable device. |  |  | 16 |
| What is the intervention meant to achieve? i.e., What mental health improvement is it targeted at (e.g., depression, anxiety, etc….)? |  | [2] | 17 |
| How long have you been using it? |  | [2] | 24 |
| What are your likes about the intervention? |  | [1, 3] | 24 |
| What things or additional services would encourage you to continually use the intervention until the desired health goal is achieved? |  | [1, 4] | 13 |
| What are your dislikes about it or any other you have used? |  | [1, 4] | 24 |
| What are the reasons you would not use or will stop using mobile health interventions? |  | [1, 4] | 24 |
| If you use a mental health app, what improvement and features, or elements will you like to see in a mental health app? |  | [3] | 19 |

**References**

[1] Peng, W., Kanthawala, S., Yuan, S., and Hussain, S. A., "A qualitative study of user perceptions of mobile health apps," *BMC public health,* vol. 16, no. 1, p. 1158, 2016, doi: <https://doi.org/10.1186/s12889-016-3808-0>. Available: <https://bmcpublichealth.biomedcentral.com/articles/10.1186/s12889-016-3808-0>.

[2] Anderson, K., Burford, O., and Emmerton, L., "Mobile health apps to facilitate self-care: a qualitative study of user experiences," *PLoS One,* vol. 11, no. 5, p. e0156164, 2016, doi: <https://doi.org/10.1371/journal.pone.0156164>. Available: <https://journals.plos.org/plosone/article?id=10.1371/journal.pone.0156164>.

[3] Stoyanov, S. R., Hides, L., Kavanagh, D. J., Zelenko, O., Tjondronegoro, D., and Mani, M., "Mobile app rating scale: a new tool for assessing the quality of health mobile apps," *JMIR mHealth and uHealth,* vol. 3, no. 1, p. e27, 2015, doi: <https://doi.org/10.2196/mhealth.3422>. Available: <https://mhealth.jmir.org/2015/1/e27>. PMID: 4376132

[4] Carolan, S. and de Visser, R. O., "Employees’ perspectives on the facilitators and barriers to engaging with digital mental health interventions in the workplace: qualitative study," *JMIR mental health,* vol. 5, no. 1, p. e8, 2018, doi: 10.2196/mental.9146. Available: <https://mental.jmir.org/2018/1/e8/?utm_source=TrendMD&utm_medium=cpc&utm_campaign=JMIR_TrendMD_0>. PMID: 29351900
