# Supplementary material for: Exploring the Barriers to and Motivators for Using Digital Mental Health Interventions Among Construction Personnel in Nigeria: Qualitative Study
Source: JMIR Form Res. 2021 Nov 9;5(11):e18969. doi: 10.2196/18969 (PMC8663629; doi:10.2196/18969)

Multimedia Appendix 3: Initial themes based on responses on barriers to using digital intervention in mental health management


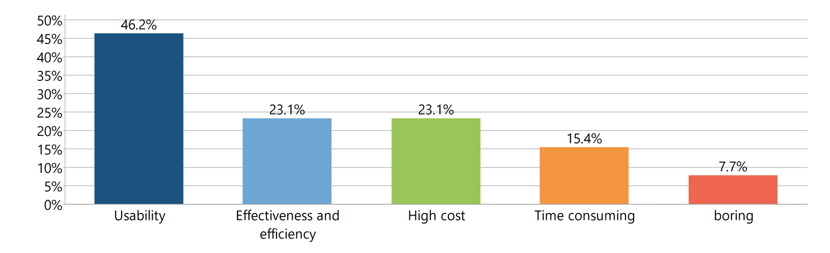

Supplement: Multimedia Appendix 3 [file formative_v5i11e18969_app3.docx]
